# Define Critical Parameters of Trastuzumab-Mediated ADCC Assays via Assay Optimization Processes, Focusing on the Impact of Cryopreserved Effector Cells on Assay Performance

**DOI:** 10.3390/cancers16132367

**Published:** 2024-06-27

**Authors:** Hanjing Peng, Yukinori Endo, Wen Jin Wu

**Affiliations:** Division of Pharmaceutical Quality Research III (OPQR III), Office of Pharmaceutical Quality Research (OPQR), Office of Pharmaceutical Quality (OPQ), Center for Drug Evaluation and Research (CDER), U.S. Food and Drug Administration (FDA), 10903 New Hampshire Avenue, Silver Spring, MD 20993, USA; hanjing.peng@fda.hhs.gov (H.P.); yukinori.endo@fda.hhs.gov (Y.E.)

**Keywords:** monoclonal antibody (mAb), antibody-dependent cellular cytotoxicity (ADCC), lactate dehydrogenase (LDH) release assay, cryopreserved effector cells, peripheral blood mononuclear cells (PBMC), natural killer (NK) cells, trastuzumab, biosimilar, human epidermal growth factor receptor 2 (HER2) positive breast cancer

## Abstract

**Simple Summary:**

The ADCC bioassays used to assess mAb-induced ADCC require continued development/refinement to meet regulatory requirements. We used trastuzumab and a lactate de-hydrogenase (LDH)-based ADCC bioassay as a model to define critical parameters of the ADCC bioassay. We find that a 4 to 24 h recovery cultivation is required to restore peripheral blood mononuclear cells (PBMCs) and natural killer (NK) cell activity toward ADCC when using cryopreserved PBMCs. Several bioassay parameters, including preparation of effector cells, E/T ratio, target cell selection, bioassay media components, and treatment time can also influence the data quality of the ADCC activity.

**Abstract:**

The mechanisms of mAb-induced ADCC have been well established. However, the ADCC bioassays used to quantify mAb-induced ADCC require continued development/refinement to properly assess and compare the potency of newly developed therapeutic mAbs and biosimilars to meet regulatory requirements. We used trastuzumab and a lactate dehydrogenase (LDH)-based ADCC bioassay as a model to define critical parameters of the ADCC bioassay, describing how several bioassay parameters, including preparation of effector cells, E/T ratio, target cell selection, bioassay media components, and treatment time can influence the data quality of the ADCC activity. We confirm that a 4 to 24 h recovery cultivation is required to restore peripheral blood mononuclear cells (PBMCs) and natural killer (NK) cell activity toward ADCC when using cryopreserved PBMCs. Furthermore, we delineated the cellular mechanisms underlying the restored ADCC activity following the recovery cultivation. We observed that CD69, an early marker of NK cell activation, was upregulated and a new subset CD56^dim^/CD16^dim^ population was dramatically increased in the recovered NK cells, which led to an increase in expression and secretion of perforin, granzyme B, and cytokine production. This study provides comprehensive technical insights into ADCC bioassay optimization to inform trastuzumab biosimilar development. The knowledge gained from this study can also be leveraged to guide bioassay development for therapeutic mAbs with ADCC as the primary mechanism of action.

## 1. Introduction

The human epidermal growth factor receptor 2 (HER2) protein is an established therapeutic target for HER2 overexpressing cancers [1,2]. Trastuzumab, an anti-HER2 monoclonal antibody (mAb), was the first approved therapeutic mAb for the treatment of breast cancer. Trastuzumab has significantly improved HER2-positive breast cancer patient outcomes and has become a standard of care for the treatment of HER2-positive breast cancer patients [3,4,5]. The success of trastuzumab has been followed by the development and approval of its biosimilars [6,7,8,9,10,11,12,13], which have helped patients gain access to these effective medications at reduced cost.

In contrast to the reference product, i.e., trastuzumab (Herceptin^®^), the FDA approves biosimilars through an abbreviated pathway, in which the analytical studies are emphasized as they provide the foundation to support structural and functional similarity between the proposed and the reference products [14]. Antibody-dependent cell-mediated cytotoxicity (ADCC) is one of the primary mechanisms of action (MOAs) mediated by trastuzumab [15,16,17]. Bioassays are used to monitor and control drug product potency/biological activity, which are important critical quality attributes (CQAs) for biosimilar assessment. Therefore, the validation of the bioassay is of great importance that ensures the data quality. Failure to establish a reliable ADCC bioassay is a significant hurdle to mAb and its biosimilars with ADCC activity as the MOA for the clinical development.

Peripheral blood mononuclear cells (PBMCs) and natural killer (NK) cells are important effector cells in the immune response to cancer [18], thus are broadly used in ADCC bioassays for testing Fc function of therapeutic mAbs. Although surrogate bioassays such as ADCC reporter assays [19] can measure the activation of engineered effector cells to monitor ADCC activities, a cytotoxicity bioassay that involves the use of PBMC-derived effector cells are often considered a more relevant bioassay that reflects the MOA of the mAbs that mediates ADCC activity. Cryopreserved PBMCs provide a consistent source of effector cells that may overcome donor-to-donor variability to minimize inconsistent data generated by ADCC bioassays. However, reports have shown that PBMCs and NK cells not only decreased recovery/viability but also impaired functions after cryopreservation [20,21,22]. The loss in NK cell functions could potentially be caused by a decrease in cell viability or 3-D migration ability [21]. Other changes in cell morphology in PBMCs after cryopreservation have also been reported [23]. While different freezing media and expansion methods have been explored to improve the recovery of cryopreservation [21,23,24,25], there are reports recommending that a “resting” cultivation period between 5 h and overnight leads to the recovery of NK cell potency [26,27,28], but this has not been sufficiently studied or reflected in most reported ADCC bioassays. In this report, the recovery of cryopreserved PBMCs and NK cells was optimized to improve the ADCC bioassay performance. Specifically, we chose to compare ADCC activity measured with NK cells isolated from cryopreserved PBMCs that are either without recovery or with 4–6 h recovery and overnight recovery period. An investigation was performed to improve the understanding of the cellular recovery mechanism.

## 2. Materials & Methods

### 2.1. Cancer Cells

SKBR-3 (RRID:CVCL_0033, human breast adenocarcinoma from mammary gland), BT-474 (RRID:CVCL_0179, human breast ductal carcinoma from mammary gland duct), MDA-MB-361 (RRID:CVCL_062, human breast adenocarcinoma from mammary gland) MDA-MB-453 (RRID:CVCL_0418, human breast adenocarcinoma from mammary gland) and NCI-N87 (RRID: CVCL_1603, human gastric carcinoma from stomach) cells were purchased from ATCC. JIMT-1 (RRID:CVCL_2077, human breast carcinoma from mammary gland) was purchased from DSMZ. All cells were maintained in a humidified 5% CO_2_ atmosphere at 37 °C. SKBR-3 was maintained in DMEM/F-12 (1:1) media containing 10% FBS; BT-474 and NCI-N87 were maintained in RPMI-1640 media containing 10% FBS; MDA-MB-361, MDA-MB-453, and JIMT-1 were maintained in DMEM media containing 10% FBS.

### 2.2. Therapeutic Monoclonal Antibody

Trastuzumab was purchased through an FDA-designated pharmacy (WEP Clinical, Morrisville, MC, USA).

### 2.3. PBMCs and NK Cells

PBMCs were isolated from human leukopak of healthy donors procured from the National Institutes of Health (NIH) (Bethesda, MD, USA) using a Ficoll-Hypaque density gradient centrifugation. ACK lysis buffer was used to remove red blood cells from PBMCs. The healthy donor information is not identifiable from human leukopak, in compliance with ethical standards. Cell strainers were used during the isolation of PBMCs to remove the cell clumps. The PBMCs were counted and frozen down at 10–50 million cells/cryovial with a freezing medium containing 90% FBS and 10% DMSO in a −80 °C freezer for short-term storage or liquid nitrogen vapor phase for long-term storage. Thawing of PBMCs was performed using a 37 °C warm bath until 80% of the ice was melted in the cryovial, then the PBMCs were transferred and resuspended into prewarmed RPMI-1640 medium containing 10% FBS and centrifuged to remove freezing medium. The PBMCs were resuspended in RPMI-1640 medium containing 10% FBS and transferred to a cell culturing dish or flask at 50 million cells/10 mL, and recovered by incubating for 4 to 24 h at 37 °C with 5% CO_2_. NK cells were isolated from unrecovered or recovered PBMCs using EasySep NK cell isolation kit (STEMCELL Technologies, cat# 17955, Vancouver, BC, Canada) according to the manufacturer’s instructions. Viability of PBMCs and NK cells was assessed using Trypan Blue Exclusion assay on a BioRad T20TM automated cell counter in the presence of Trypan Blue to assess live cell numbers.

### 2.4. LDH ADCC Assay

The ADCC bioassay was conducted using the LDH-Glo™ Cytotoxicity Assay kit (Promega, Cat# J2381, Madison, WI, USA) according to the manufacturer’s instructions. Briefly, target cells were plated at 5000 cells/well in a tissue-culture-treated 96-well plate with RPMI-1640 medium containing 1% BSA (Sigma Aldrich, Cat# A7030-10G, St. Louis, MI, USA). Trastuzumab was added at the indicated concentrations. NK cells were added at the indicated E/T ratios and the plates were incubated in a humidified 5% CO_2_ atmosphere at 37 °C. At the end of the incubation, 10 µL of conditioned medium was taken out from each well, diluted with LDH storage buffer (200 mM Tris-HCl (pH 7.3), 10% Glycerol, 1% BSA) in a round-bottomed 96-well dilution plate, and mixed with the LDH assay enzyme cocktail and fluorogenic substrate mixture in a flat-bottomed 96-well opaque assay plate. The plate was incubated on a shaker for 30–40 min and luminescence was measured with a Promega GloMax^®^ Discover Microplate Reader. Specific cell killing was reported as fold increase in luminescence (fold increase in luminescence = (experimental LDH release luminescence-medium background luminescence)/medium background luminescence.

### 2.5. ADCC Reporter Assay

Induction of NFAT signaling in effector cells was measured using the ADCC Reporter Bioassay kit (Promega, Cat# G7010). The ADCC reporter bioassay was performed in a 96-well plate format according to manufacturer’s instructions. Briefly, the target cells, SK-BR-3, were seeded in a flat-bottomed 96-well opaque plate at 5000 cells/well in 100 µL volume using the ADCC Bioassay buffer as described in the manufacturer’s kit. The serial dilutions of trastuzumab were added into each well the next day. ADCC effector cells were thawed and prepared according to the manufacturer’s protocol and added to the target cells at an effector-to-target ratio of 6:1. After 5–6 h of incubation in the cell culture incubator, the Bio-Glo™ Luciferase Assay Reagent was added to the plates and luminescence was measured with a Promega GloMax^®^ Discover Microplate Reader. NFAT induction in effector cells was reported as fold of induction = RLU (induced—background)/RLU (no antibody control—background).

### 2.6. Western Blotting Analysis

For HER2 expression analysis, SKBR-3, BT-474, NCI-N87, MDA-MB-361, MDA-MB-453, and JIMT-1 cells were lysed at 5 × 10^5^ cells/100 µL NP40 lysis buffer on ice for 30 min. For CD16A expression, NK cells were lysed in Cell Signaling lysis buffer (Cat# 9803) on ice for 30 min. For perforin and granzyme B expression in NK cells in a mono- or coculturing with SKBR-3 w/wo trastuzumab treatment, SKBR-3 cells were plated in a 24-well plate at 1 × 10^5^ cells/well with RPMI-1640 medium containing 1% BSA (Sigma Aldrich, Cat# A7030-10G). Trastuzumab was added at 0.1 µg/mL. NK cells were added at 5 × 10^5^ cells/well and the plates were then incubated for 24 h in a humidified 5% CO_2_ atmosphere at 37 °C. At the end of the incubation, cells were collected and lysed in Cell Signaling lysis buffer for 30 min on ice. The cell lysates were centrifuged and the harvested protein supernatants were boiled with SDS loading buffer at 95 °C for 5 min then subjected to Western blot analysis. ImageJ software, version 1.54j (NIH, Bethesda, Rockville, MD, USA) was used for densitometry of Western blotting. The following primary antibodies were used for Western blotting analysis: HER2 (Cell Signaling, Cat# 2165S, RRID:AB_10692490), Perforin (Cell Signaling, Cat# 62550, RRID:AB_3095060), Granzyme B (Cell Signaling, Cat# 4275, RRID:AB_2114432), CD16A (Cell Signaling, Cat# 19731, RRID:AB_2798825), GAPDH (Sigma Aldrich, Cat# G8795, RRID:AB_1078991), and β-Actin (Sigma Aldrich, Cat# A1978, RRID:AB_476692).

### 2.7. Flow Cytometry

A FlowX Human NK Cell Phenotyping Flow Cytometry Kit (R&D Systems, Cat# FMC033, Minneapolis, MN, USA) was performed to assess NK cell phenotype using flow cytometry. Briefly, PBMCs or NK cells (5 × 10^5^ cells per sample) were washed with Staining Buffer (R&D Systems, Cat# FC001), Fc-blocked with BD Pharmingen™ Human BD Fc Block™ (BD, Cat# BDB564219) at 1 µg/10^6^ cells in 100 µL of Staining Buffer for 10 min at room temperature, and incubated with 5 µL of each antibody or isotype control per 10^6^ cells for 30–45 min at room temperature in the dark. At the end of the incubation, the cells were washed and resuspended in 1 mL Staining Buffer and analyzed using an LSR Fortessa flow cytometer (BD Bioscience, San Jose, CA, USA).

For analysis of surface expression of CD69, Fc-blocked NK cells (5 × 10^5^ cells per sample) were incubated with CD69 Mouse anti-Human, FITC, Clone: FN50 (2.5 µg per 10^6^ cells, BD Biosciences Cat# BDB555530) or FITC Mouse IgG1ĸ Isotype control (2.5 µg per 106 cells, BD Biosciences Cat# BDB555748) for 30–45 min, washed, resuspended, and analyzed by flow cytometry.

For analysis of surface expression of CD16, Fc-blocked NK cells (5 × 10^5^ cells per sample) were incubated with CD16 mouse anti-human, PE-Cy5, Clone: 3G8 (2.5 µg per 10^6^ cells, BD Biosciences Cat# BDB555408), or PE-Cy5 Mouse IgG1ĸ Isotype control (2.5 µg per 106 cells, BD Biosciences Cat# BDB555750) for 30–45 min, washed, resuspended, and analyzed by flow cytometry.

### 2.8. ELISA Assays

Secretion of perforin, granzyme B, IFNγ, and TNFα in conditioned media from mono- or coculturing of NK and SKBR-3 cells were measured using the following ELISA assays: Human Perforin ELISA Kit (PRF1) (Abcam, Cat# ab46068, Waltham, MA, USA), Human Granzyme B Quantikine ELISA Kit (R&D Systems, Cat# DGZB00), Human IFN-gamma Quantikine ELISA Kit (R&D Systems, Cat# DIF50C), and Human TNF-alpha Quantikine ELISA Kit (R&D Systems, Cat# DTA00D). Briefly, SKBR-3 cells were plated in a 24-well plate at 1× 10^5^ cells/well with RPMI-1640 medium containing 1% BSA (Sigma Aldrich, Cat# A7030-10G). Trastuzumab was added at 0.1 µg/mL. NK cells were added at 5 × 10^5^ cells/well and the plates were incubated for 24 h in a humidified 5% CO_2_ atmosphere at 37 °C. At the end of the incubation, conditioned cell culturing medium in each well was collected and centrifuged to remove cells. The supernatant was used for the ELISA assays according to instructions provided by the manufacturers.

### 2.9. Knockdown of HER2 in SKBR-3 Cells

A total of 500 nM siRNA was transfected into 1 × 10^6^ SKBR-3 cells using 4D-Neucleofector (Lonza bioscience, Morrisville, NC, USA). Then, 48 h post transfection, the cells were used for ADCC reporter and LDH ADCC assays. The HER2 knockdown efficiency was evaluated by Western blot analysis after 72 h post transfection. The following siRNAs were purchased from Dharmacon (Horizon discovery): ON-TARGETplus SMARTpool human HER2 siRNA (cat# L-003126-00-0005, siRNA target sequence: UGGAAGAGAUCACAGGUUA, GAGACCCGCUGAACAAUAC, GGAGGAAUGCCGAGUACUG, GCUCAUCGCUCACAACCAA), and ON-TARGETplus nontargeting pool control siRNA (cat# D-001810-10-20, siRNA target sequence: AGACAAUGCUGUACGGAAU, GGCUAAAGCUCCAGGCGUU, CAAAGGAUGUCAUUCGUAA, AAUAAAGAGCAGUCGCAAA).

### 2.10. Statistical Analysis

GraphPad Prism, version 10.2.3, was used for statistical studies. Statistical significance was determined by one-way or two-way ANOVA with Tukey’s post hoc test or Šídák’s post hoc test (*, *p* value < 0.05; **, *p* value < 0.01; ***, *p* value < 0.001; ****, *p* value < 0.0001). Data are expressed as mean ± SD.

## 3. Results

### 3.1. Influence of Cryopreservation and the Recovery Cultivation on PBMCs and NK Cells

Due to the lack of readily available fresh blood donations, cryopreserved PBMCs are often used for ADCC bioassays, which provides a consistent source of the effector cells. However, the low signal intensity of cryopreserved PBMCs or NK cells isolated from PBMCs has been identified as a common problematic issue that may impact the data quality. It is believed that cryopreservation of PBMCs and NK cells may affect their ability to mediate cytotoxicity on the target cells, leading to inconsistent ADCC data.

**Figure 1:**
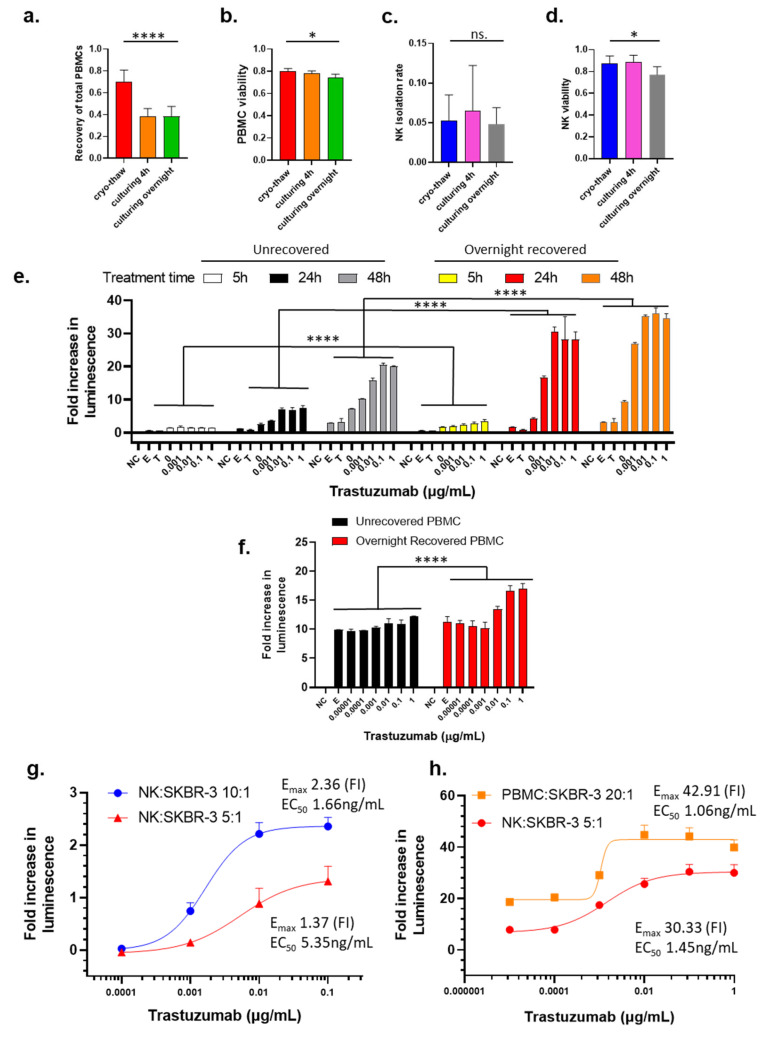
Influence of effector cell preparation, choice of effector cell, and E/T ratio on the measurement of trastuzumab-mediated ADCC using LDH ADCC assay. a–d. Influence of cryopreservation and the recovery cultivation time (4 h to overnight in RPMI-1640 with 10% FBS) on the recovery rate (**a**), viability of recovered PBMCs (**b**), the rate of NK cell isolation from the PBMCs (**c**), and the viability of isolated NK cells (**d**). Data were analyzed with GraphPad Prism using one-way ANOVA mixed-effects statistical analysis based on matched rows and recovery time as repeated measure with Tukey’s post hoc test. Overall *p* values for Figure 1a–d are <0.0001, 0.0126, 0.2553 (no statistical significance among three groups), and 0.0185, respectively; * *p* < 0.05 and **** *p* < 0.0001 (**e**) ADCC activity of trastuzumab with NK cells from immediately thawed PBMCs or overnight recovered PBMCs as effector cells with E/T ratio 20:1 measured after treatment for 5 h, 24 h, or 48 h. (**f**) ADCC activity of trastuzumab with immediately thawed or overnight recovered PBMCs as effector cells with E/T ratio 20:1 after treatment for 24 h. Data were analyzed with GraphPad Prism using two-way ANOVA with Šídák’s multiple comparisons test. Overall *p* value < 0.0001 for unrecovered compared to overnight recovered PBMCs. For (**e**,**f**), SKBR-3 cells were treated with trastuzumab (0–1 µg/mL) and NK cells, and killing of SKBR-3 was measured using the LDH ADCC assay. NC stands for the no-cell or media-only control, E stands for the effector-cell-only control, and T stands for the target-cell-only control. Data were analyzed with GraphPad Prism using two-way ANOVA, and *p* values are <0.0001. (**g**) Comparing E_max_ and EC_50_ values of LDH ADCC activity between different E/T ratio with NK cells as effector cells. (**h**) Comparing E_max_ and EC_50_ of LDH ADCC activity between PBMCs (E/T ratio: 20:1) and NK cells (E/T ratio: 5:1) as effector cells. Bars represent average values, with the standard deviation shown as brackets.

To determine if cryopreservation followed by recovery cultivation may impact the PBMCs and NK cells, we monitored the cell viability, PBMCs total recovery rate, and NK cell isolation rate after thawing the cells from cryopreservation and after recovery cultivation for 4 h and overnight. As compared to the noncryopreserved PBMCs that were freshly added in the cryovial, the cryopreserved PBMCs have a 69.1% recovery rate immediately after thawing (Figure 1a). After 4 h in recovery cultivation, the total PBMCs further decreased to 38.9%, which remained comparable to the PBMCs subjected to overnight cultivation (Figure 1a). The viability of PBMCs slightly decreased from 80.0% to 74.6% after overnight cultivation (Figure 1b). These data suggest that the total recovery of the PBMCs following the initial thawing from cryopreservation was biphasic with an initial phase with a decrease in total recovery at 4 h post thaw and then a secondary phase that does not further decrease the total recover rate at 24 h post thaw. However, the viabilities of recovered PBMCs were only slightly affected by culturing time.

The cryopreservation followed by recovery cultivation does not affect the isolation rate of NK cells from the PBMCs, as shown in Figure 1c. There was a slight decrease in the viability of isolated NK cells between cryo-thaw and overnight cultivation (87.2% and 77.0%, respectively), whereas there was essentially no difference observed between cryo-thaw and 4 h recovery cultivation (87.2% and 88.5%, respectively) (Figure 1d).

### 3.2. Influence of Cryopreservation and Recovery Cultivation of PBMCs and NK Cells, Treatment Time, E/T Ratio, and Type of Effector Cells on ADCC Activity Measurement

Among various reported assays to measure ADCC (radioactive chromium release assay [29], DELFIA-EuTDA Cytotoxicity Assay [30,31], lactate dehydrogenase (LDH) release assay [32,33], flow cytometry assays [34,35], and HiBiT target cell killing assay [36]), the LDH release assay was selected to monitor for ADCC activity for this study because this assay does not require a pretreatment or reagent loading step. In this assay, LDH released into the media from damaged target cells can be quantified via luminescence and the readouts can be compared to monitor for changes in cell damage between cultivation conditions.

To test if overnight recovery cultivation potentiates cytotoxic activity of cryopreserved effector cells, we compared dose-dependent ADCC activity of trastuzumab with effector cells that had been subjected to overnight recovery cultivation to effector cells that were not exposed to recovery cultivation. To avoid donor-to-donor or batch-to-batch variability, unless otherwise stated, PBMCs used for direct comparison within each experiment throughout this project were always from the same donor and cryopreserved from the same leukopak. Data were analyzed using two-way ANOVA with Tukey’s post hoc test in GraphPad Prism. NK cells isolated from PBMCs that underwent the overnight recovery cultivation showed significantly enhanced killing as compared to NK cells isolated immediately from thawed cryopreserved PBMCs (Figure 1e, black bars compared to red bars, *p* < 0.0001 for all trastuzumab treatment concentrations). Next, the ADCC activity of trastuzumab using immediately thawed and overnight recovery cultivated PBMCs was monitored to reveal that the PBMCs subjected to recovery cultivation had a significantly higher signal than the immediately thawed PBMCs (Figure 1f, *p* < 0.0001 for trastuzumab concentrations at 1 µg/mL and 0.1 µg/mL; *p* = 0.0089 for trastuzumab concentration at 0.01 µg/mL in a two-way ANOVA with Šídák’s multiple comparisons test). These data clearly support that recovery cultivation of cryopreserved PBMCs used as effector cells and NK cells can significantly improve the luminescence signal that is used for monitoring the cytotoxic response with ADCC assays.

Treatment time is an important parameter to consider. The initiation of the ADCC mechanism is known to have rapid response for the activation of effector cells, which can be detected within 4 h [19]. However, the capability to detect cell killing may take a longer period to reach the required number of dead cells. When exposing cells to trastuzumab for 5 h, the cell killing signals were detected at a very low level (Figure 1e, yellow bars), but extending the treatment time to 24 h drastically increased the cell killing when the NK cell from the overnight recovery cultivation PBMCs were used as effector cells (Figure 1e, red bars). After 48 h of treatment, the fold increase in luminescence observed was only slightly higher than that for 24 h treatment (Figure 1e, orange bars), indicating that the 24 h treatment was adequate for this assay. However, NK from unrecovered PBMCs as effector cells caused limited cell killing at 5 h post trastuzumab treatment (Figure 1e, white bars), and prolonging the treatment time to 24 h only resulted in about 30% of maximum killing of overnight recovered effector cells used (Figure 1e, black bars). Moreover, the signal further increased between the 24 h and 48 h exposure times (Figure 1e, gray bars), albeit the killing was still much lower than that measured with overnight recovered effector cells (Figure 1e, orange bars). However, LDH assay with 24 h treatment gave much higher signal-to-noise ratio than 5 h and 48 h (Figure S1). Therefore, treatment time was selected to be within a 24 h period.

The effector/target (E/T) ratio is an important parameter for ADCC assays because higher E/T ratio provides higher cytotoxicity according to previous reports [37,38], while E/T ratio is also limited by the availability of effector cells in the experimental conditions. Our data show that when the E/T ratio increased from 5:1 to 10:1, the E_max_ increased from 1.37 to 2.36, while the potency of trastuzumab also increased with a decreased EC_50_ 5.35 to 1.66 ng/mL (Figure 1g). Although NK cells are believed to play a major role in the ADCC MOA and the number of NK cells represent 5–20% of PBMCs [39], the use of PBMCs at an E/T ratio of 20:1 and NK cells at an E/T ratio of 5:1 showed comparable results in terms of E_max_ and EC_50_ (Figure 1h). It should be noted that the NK cells used for the experiments shown in Figure 1g,f were from different donors, indicating the donor-to-donor difference that contribute variability of ADCC results.

### 3.3. The Recovery Cultivation Condition Influences NK Cells Mediated ADCC Activity

**Figure 2:**
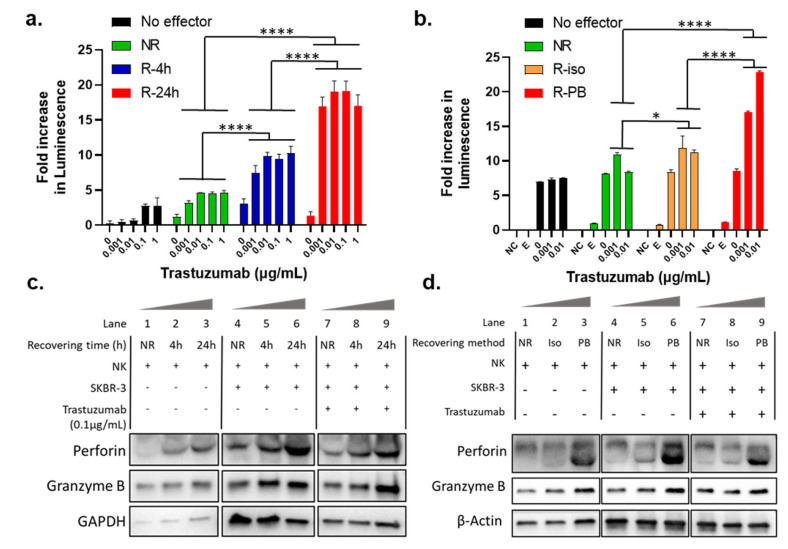
Influence of the recovery culturing time and method on LDH ADCC assay and cellular expression of granulation proteins. (**a**) Comparison of ADCC activity of trastuzumab with NK cells isolated from PBMCs recovered for different times 0 h (NR), 4 h, or 24 h. Data were analyzed with GraphPad Prism using two-way ANOVA with Tukey’s multiple comparisons test. **** *p* < 0.0001; (**b**) Comparison of trastuzumab induced ADCC activity using NK cells that underwent overnight recovery after isolation from thawed cryopreserved PBMCs (R-iso for “recovered after isolation”), and NK cells isolated from the overnight recovered PBMCs (R-PB). Data were analyzed with GraphPad Prism using two-way ANOVA with Tukey’s multiple comparisons test. **** *p* < 0.0001. SKBR-3 cells were treated with trastuzumab (0–0.01 µg/mL) and NK cells with E/T ratio 5:1 for 24 h, and killing of SKBR-3 was measured with LDH ADCC assay; (**c**) Representative antiperforin, antigranzyme B, and Anti GAPDH immunoblot in NK cells isolated from unrecovered (NR) PBMCs or from PBMCs after recovery cultivation for different time points (4 h, 24 h) with E/T ratio 5:1. Lanes 1–3, 4–6, and 7–9 show expression levels for NK cells after incubation for 24 h hours with media only, with SKBR-3 only, and with SKBR-3 and trastuzumab, respectively. All lanes were run in the same gel, and images were broken into three different treatment groups; (**d**) Representative antiperforin, antigranzyme B, and Anti β-actin immunoblot in NK cells isolated from unrecovered PBMCs (NR), and NK cells recovered for 24 h after isolation from cryopreserved PBMCs (Iso), and NK cells isolated from PBMCs after recovery cultivation for 24 h (PB) with E/T ratio 5:1. Lanes 1–3, 4–6, and 7–9 show expression levels for NK cells after incubation for 24 h hours with media only, with SKBR-3 only, and with SKBR-3 and trastuzumab, respectively. All lanes were run in the same gel, and images were broken into three different treatment groups.

To further understand the mechanism(s) contributing to the restoration of NK cell activity during the recovery cultivation, we investigated the influence of culturing condition. Firstly, we tested the influence of the recovery cultivation time on the potentiation of ADCC function of the NK cells. Specifically, we compared the ADCC activity of trastuzumab with NK isolated from immediately thawed PBMCs (NR stands for “not recovered”), and NK isolated from PBMCs that underwent recovery cultivation for 4 h (R-4 h) or 24 h (R-24 h). All the PBMCs and NK cells were prepared from the cryopreserved PBMCs from the same donor. The LDH ADCC assay results in Figure 2a showed that NK cells from PBMCs recovered for 4 h showed stronger ADCC activity than unrecovered, but lower ADCC than those recovered for 24 h (*p* < 0.0001). This result demonstrates that the potentiation of NK activity by the recovery culturing of cryopreserved PBMCs was a time-dependent process.

In the experiments described in previous sections, the recovery culturing was performed with the PBMCs, and the NK cells that were isolated from the recovery cultivated PBMCs. As PBMCs contain a variety of cell types, we were interested to test if other cells in the PBMC population play a role in NK activation. Specifically, we tested if the recovery culturing still potentiates the NK cells when other cells in the PBMC population were excluded from the recovery cultivation condition. We compared the dose-dependent ADCC activity of trastuzumab in the absence of NK (No effector), with unrecovered NK cells (NR), NK cells that underwent overnight recovery after isolation from thawed cryopreserved PBMCs (R-iso for “recovered after isolation”), and NK cells isolated from the overnight recovered PBMCs (R-PB). To help in understanding this, Figure S1 shows a flow scheme of two different recovery culturing method. As shown in Figure 2b, NK cells isolated from overnight recovered PBMCs (R-PB) showed much stronger killing signal toward ADCC than NR and R-iso (*p* < 0.0001). These data indicate that the existence of other cells in the PBMCs may help with the potentiation of the NK cells towards ADCC activity. Therefore, using NK cells isolated from the recovered PBMCs is a better approach to prepare effector cells.

### 3.4. Recovery Cultivation Influences the Expression Levels of Perforin and Granzyme B in NK Cells

Perforin and granzyme B are key lytic proteins synthesized by NK cells that play a key role in the ADCC process. Therefore, we were interested to compare the protein expression levels of perforin and granzyme B in NK cells before and after recovery culturing. Specifically, we performed Western blot analysis for the NK cells incubated with or without SKBR-3 as target cells, treated with or without 0.1 µg/mL trastuzumab. These NK cells were either isolated from unrecovered PBMCs (NR) or from PBMCs that underwent recovery cultivation for 6 h or 24 h. As shown in Lanes 1–3, 4–6, and 7–9 in Figure 2c, both perforin and granzyme B protein levels showed time-dependent increases during recovery cultivation in all three treatment groups, which was consistent with the time-dependent enhancement in ADCC activity shown in Figure 2a. These data suggest that the NK cell potentiation during the recovery culturing period was due to higher expression of key lytic proteins and the upregulation was time-dependent over a 24 h time period.

In Figure 2b, we show that NK cells isolated from the overnight recovery cultivated PBMCs (R-PB) provided higher ADCC activity as compared to the NK cells did not undergo recovery cultivation (NR), or NK cells recovered without other cells in the PBMC population (R-iso). As a proof of concept, we tested lytic protein expression levels using Western blot in NK cells from the abovementioned treatments. As expected, Lanes 1–3, 4–6, and 7–9 in Figure 2d also showed higher perforin and granzyme B protein expression in all three groups in PB than NR and Iso. This evidence supports the idea that NK cell potentiation during the recovery culturing period was, indeed, due to higher expression of perforin and granzyme B. Note that lanes 4–9 in Figure 2c,d include both NK and SKBR-3 cell lines. However, SKBR-3 does not interfere with the WB results since there is no expression of either perforin or granzyme B in SKBR-3 cells.

**Figure 3:**
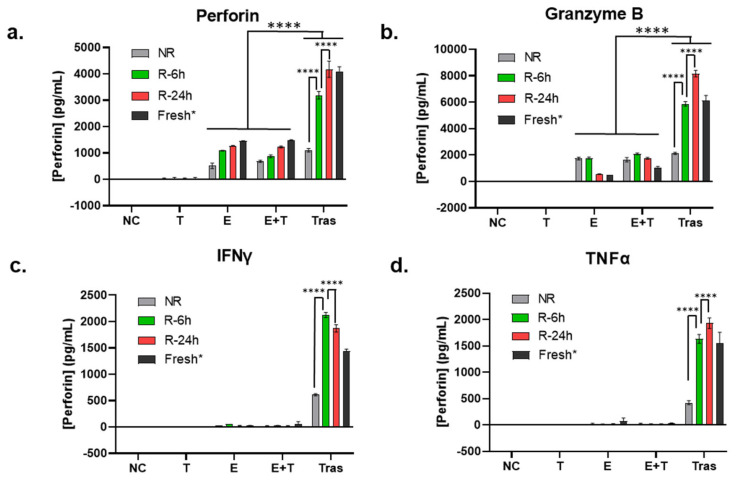
Influence of the recovery culturing time and method on the extracellular release of granulation proteins and cytokines secretion. (**a**–**d**) Comparison of the secretion levels of perforin (**a**), granzyme B (**b**), TNFα (**c**), and IFNγ (**d**) using ELISA in the 24 h cell culture media of the target cell SKBR-3 (T), the effector cells NK (E), coculture of SKBR-3 and NK (E+T), and trastuzumab-treated coculture of SKBR-3 and NK (Tras) with E/T ratio 5:1. Data in this figure were analyzed with GraphPad Prism using one-way ANOVA analysis with Tukey’s post hoc test (**** *p* < 0.0001). * Fresh NK cells were prepared from a different donor; thus, they were not included in statistical analysis.

In addition to Western blot analysis of endogenous perforin and granzyme B, we also tested the secreted perforin and granzyme B in the cell culture media after 24 h of treatment using ELISA. In this experiment, NK cells from unrecovered (NR) and recovered (R-4 h and R-24 h) PBMCs were compared. NK cells isolated from fresh PBMCs from a different donor and not cryopreserved were included as a control (but not included in statistical analysis). As shown in Figure 3a (perforin) and Figure 3b (granzyme B), unlike protein levels measured using whole cell lysates, the secreted levels of perforin and granzyme B from the coculturing with trastuzumab group (Tras) were much higher than that in effector only (E) and coculture of NK and SKBR-3 cells (E+T) (*p* < 0.0001), indicating that trastuzumab was essential to initiate ADCC, or the cell killing process. On the other hand, the levels of secreted perforin and granzyme B in the Tras group were also clearly dependent on the recovery cultivation time (NR < R-4 h < R-24 h, *p* < 0.0001), and the secreted perforin and granzyme levels from NK cells isolated from recovery cultured PBMCs were comparable to NK cells isolated from fresh PBMCs.

### 3.5. Recovery Cultivation Is Essential for NK Cells to Secret Cytokines to Mediate ADCC Activity

In addition to the lytic proteins, inflammatory cytokines produced by activated NK cells, such as TNFα and IFNγ, are also important contributing factors for NK cell cytotoxic functions [40]. Therefore, TNFα and IFNγ in the cell culture media collected for measuring perforin and granzyme B levels (the same media as that used for Figure 3a,b) were quantified using ELISA. As shown in Figure 3c,d, these two cytokines remained undetectable in the effector-only (E) and coculturing without trastuzumab (E+T) groups. In contrast, a significant amount of TNFα and IFNγ was detected in the coculturing with trastuzumab (Tras) group. Within the Tras group, NK cells isolated from immediately thawed PBMCs (NR) had a significantly lower TNFα and IFNγ production than the NK cells isolated from recovery cultured PBMCs (R-6 h and R-24 h, *p* < 0.0001), which were at levels comparable to NK cells isolated from fresh PBMCs (Fresh) (Figure 3c,d).

### 3.6. CD16/FcγRIII Expression Is Downregulated during the Recovery Culturing of NK Cells

**Figure 4:**
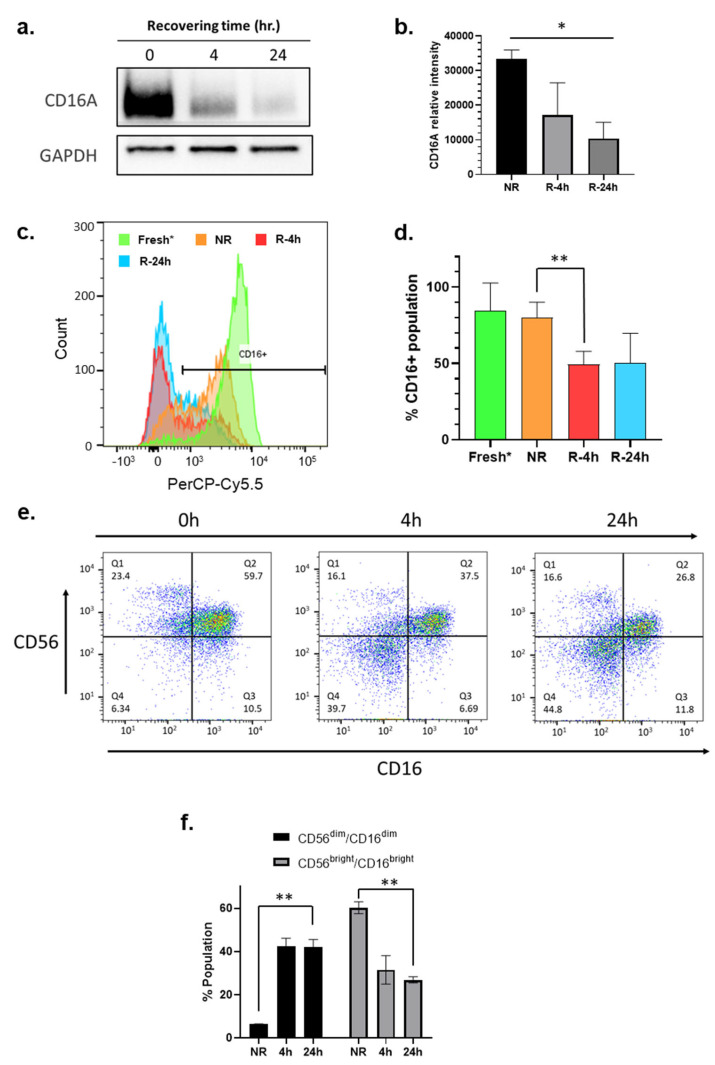
The recovery culturing downregulates expression of CD16 and CD56 on NK cells. (**a**) Representative immunoblot of CD16A (FcγRIIIa) in the whole cell lysates of NK cells isolated from unrecovered (NR) PBMCs or from PBMCs after recovery cultivation for different time points (4 h, 24 h); (**b**) Quantitation of three independent CD16A immunoblots; (**c**) Flow cytometry analysis of surface CD16 (FcγRIII) on live NK cells isolated from fresh (neon green), thawed cryopreserved (NR, orange) PBMCs, or from PBMCs after recovery cultivation for different time points 4 h (red) and 24 h (blue). Data in this figure were analyzed with GraphPad Prism using one-way ANOVA mixed—effects statistical analysis based on matched rows and recovery time as repeated measure with Tukey’s post hoc test. *p* = 0.0279; (* *p* < 0.05). (**d**) Quantitation of replicated flow cytometry results shown in (**c**) *p* = 0.0708; (**e**) Flow cytometry analysis of NK cells isolated from unrecovered (NR) PBMCs or from PBMCs after recovery cultivation for different time points (4 h, 24 h); (**f**) Quantitation of replicated flow cytometry results shown in (**e**) *p* = 0.0037 (** *p* < 0.01) (time factor) within CD56^dim^/CD16^dim^, *p* = 0.0040 (** *p* < 0.01) (time factor) within CD56^bright^/CD16^bright^. Fresh NK cells were prepared from a different donor.

The CD16A, also known as FcγRIIIa, is a receptor protein predominantly expressed by NK cells and is known to play a crucial role in mediating ADCC [41]. To determine if recovery cultivation impacts the expression level of CD16A, immunoblotting was performed to reveal a time-dependent downregulation of CD16A protein expression during the 24 h recovery cultivation period (Figure 4a,b). A 30% decrease in CD16A expression was observed in the NK cells isolated from PBMCs subject to a 4 h and 24 h recovery cultivation period compared to NK cells isolated from immediately thawed PBMCs (NR). To support these findings, flow cytometry was performed as an orthogonal method to confirm the reduction in CD16A on the NK cell plasma membrane (Figure 4c). Similar to the immunoblotting data, flow cytometry revealed a decrease in CD16A protein expression on NK cells isolated from PBMCs subject to 4 or 24 h recovery cultivation period as compared to NK cells from fresh and immediately thawed PBMCs (Figure 4c). Around 50% of NK cells isolated from 24 h recovery cultured PBMCs had surface expression of CD16, compared to approximately 80% in NK cells isolated from immediately thawed PBMCs (Figure 4c). When comparing the histograms of CD16 expression in NK cells isolated from fresh PBMCs, immediately thawed PBMCs, 4 h, and 24 h recovered PBMCs, we observed a dynamic shift of NK population from bright to dim, and from dim to probably negative (Figure 4c). This observation suggested that a concerted loss of CD16 on NK cell surface occurred during the freeze–thaw and the recovery cultivation period.

### 3.7. Phenotyping Analysis Reveals the Formation of CD56^dim^/CD16 ^dim^ Subpopulation in NK Cells during the Recovery Cultivation Period

Flow cytometry revealed that the NK cells from cryopreserved PBMCs before and after the recovery cultivation had downregulated surface CD56 expression (Figure 4e). NK cells are known to be the CD3^−^ subpopulation in PBMCs. Figure 4e,f showed that downregulation of CD56 and CD16 during the recovery cultivation period caused a dramatic increase in the formation of a new CD56^dim^/CD16^dim^ subpopulation of NK cells. The simultaneous downregulation of CD56 and CD16 might be an indicator of NK cell activation.

### 3.8. CD69 Expression Is Dramatically Upregulated on the Surface of NK Cells during the Recovery Cultivation Period

**Figure 5:**
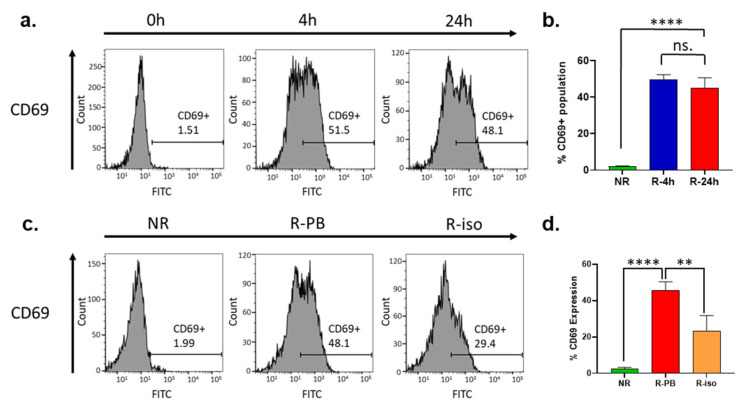
Phenotyping by flow cytometry indicates activation of NK cells by upregulation of CD69 during the recovery cultivation. (**a**) Flow cytometry analysis of surface CD69 expression in NK cells isolated from unrecovered (NR) PBMCs or from PBMCs after recovery cultivation for different time points (4 h, 24 h); (**b**) Quantitation of replicated flow cytometry results shown in (**a**). Data in this figure were analyzed with GraphPad Prism using one-way ANOVA analysis with Tukey’s post hoc test. **** *p* < 0.0001 and ** *p* < 0.01; (**c**) Flow cytometry analysis of surface CD69 expression in NK cells isolated from unrecovered PBMCs (NR) and NK cells recovered for 24 h after isolation from cryopreserved PBMCs (Iso), and NK cells isolated from PBMCs after recovery cultivation for 24 h (PB); (**d**) Quantitation of replicated flow cytometry results shown in (**c**).

During the recovery cultivation period, the expression of other cell surface markers such as CD14, CD25, and CD107a was found to be mostly unchanged (Figure S3). However, CD69 expression was dramatically upregulated in the NK cells (Figure 5a). CD69 is a classical early marker of lymphocyte activation, including NK cells, due to its rapid appearance on the surface of the plasma membrane after stimulation [42]. The CD69 bearing cells increased from <2% to approximately 50% (*p* < 0.0001) after the PBMCs were exposed to a 4 h recovery cultivation period (Figure 5a,b). On the other hand, the increase in cell surface CD69 in NK cells isolated from the overnight recovered PBMCs (R-PB) was greater than that in NK cells recovered after isolation (R-iso) (Figure 5c,d). These differences were consistent with the enhancement in ADCC activity shown in Figure 2a,b. The isotype control experiment showed complete Fc blocking (Figure S4). Therefore, we believe that the upregulation of CD69 leads to the activation of NK cells and simultaneous downregulation of CD56 and CD16, which in turn promotes expression and secretion of perforin and granzyme B, leading to enhanced ADCC activity.

### 3.9. Choice of Target Cell Lines in the LDH ADCC Assay and ADCC Reporter Assay

**Figure 6:**
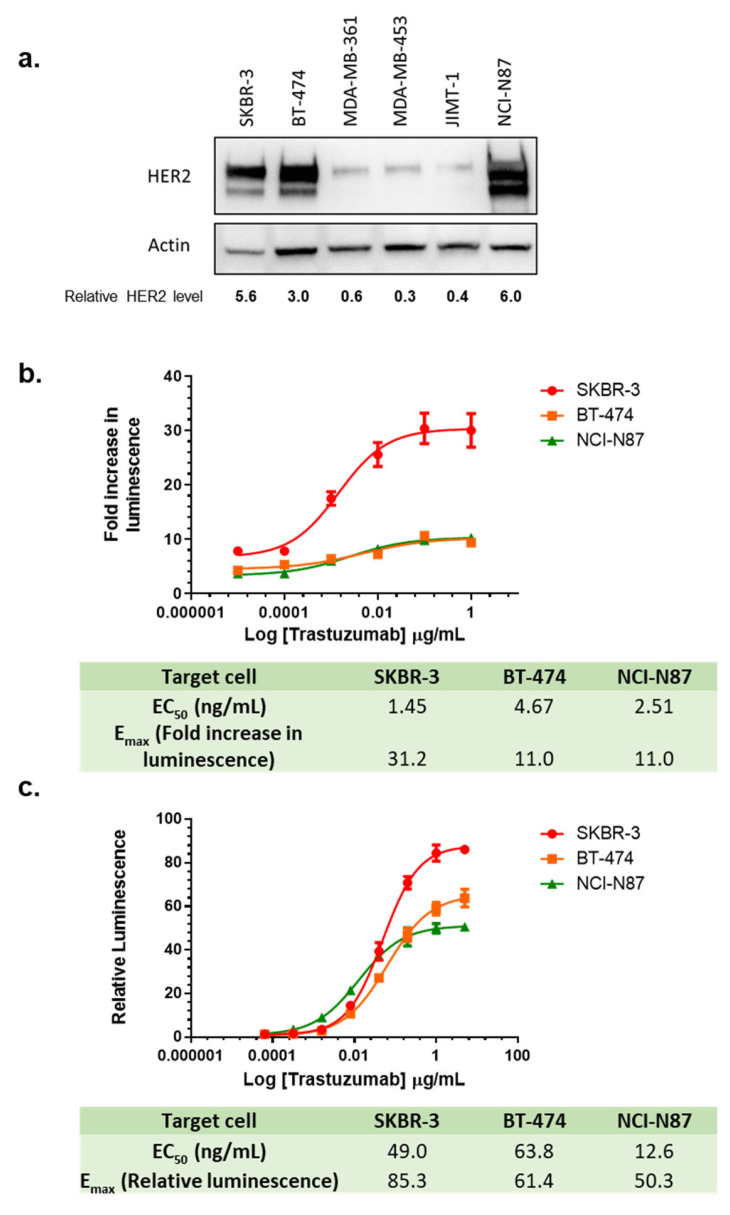
Choice of target cell lines in the LDH ADCC assay and ADCC reporter assay. (**a**) Representative immunoblots using lysates from HER2-positive cell lines; (**b**) Quantitation of the immunoblot; (**c**) Dose-dependent curves of trastuzumab-mediated LDH ADCC assay using SKBR-3, BT-474, and NCI-N87 cells. Target cells were treated with trastuzumab (0–1 µg/mL) and NK cells with E/T ratio 5:1 for 24 h and killing of SKBR-3 was measured with LDH ADCC assay; (**c**) Dose-dependent curves of trastuzumab-mediated ADCC activity using ADCC reporter assay in SKBR-3, BT-474 and NCI-N87 cells. Target cells were treated with trastuzumab (0–5 µg/mL) and Promega ADCC reporter cells with E/T ratio 6:1 and luminescence was measured after 6 h.

In addition to effector cells preparation, the selected target cell line is another critical component for ADCC assays. Trastuzumab was approved for treatment of HER2-overexpressing breast cancer and HER2-overexpressing metastatic gastric or gastroesophageal junction adenocarcinoma. To select the target cell line, immunoblotting was performed to identify the HER2 expression level from a series of breast cancer cell lines [SKBR-3, BT-474, MDA-MB-361, MDA-MB-453, JIMT-1] and gastric cancer cell line [NCI-N87] (Figure 6a). Based on the high levels of HER2 expression, SKBR-3, BT-474, and NCI-N87 were chosen as the target cells for the ADCC assays (Figure 6a). Using the LDH release assay, a clear dose response was observed for trastuzumab in all three cell lines, with the SKBR-3 cell line having a much higher signal-to-noise (S/N) ratio or E_max_, while BT-474 and NCI-N87 showed lower but similar E_max_ (Figure 6b). To test if the changes in the levels of HER2 expression in the same cell line affect ADCC activity, we knocked down HER2 in SKBR-3 with siRNA (Figure S5a), and compared ADCC effect in the cells treated with HER2 siRNA and control siRNA (scrambled). We found that E_max_ in the LDH assay did not seem to be sensitive to HER2 knockdown, while the EC_50_ showed a significant increase (Figure S5b).

The ADCC reporter assay is a surrogate assay often utilized to demonstrate ADCC biological activities in drug development [19]. We compared the ADCC reporter assay to the LDH ADCC assay by monitoring dose-dependent activity using SKBR-3, BT-474, and NCI-N87 as target cells (Figure 6c). Consistent with our LDH ADCC assay, SKBR-3 provided the highest S/N ratio among these three target cell lines, indicating the highest activation of NFAT pathway in the reporter effector cells. In contrast, the S/N ratio in BT-474 was lower than in SKBR-3 but higher than in NCI-N87. These results demonstrated that SKBR-3 showed the highest S/N ratio in all three HER2 overexpressing cells in both LDH ADCC assay and the ADCC reporter gene assay, providing better data quality when evaluating ADCC activities of trastuzumab. When testing the influence of HER2 knockdown in the ADCC reporter assay, we found that E_max_ values from the ADCC reporter assay were sensitive to HER2 knockdown (Figure S5c). When plotting E_max_ of the ADCC reporter assay against HER2 expression levels reported in the literature in SKBR-3, BT-474, and NCI-N87 [43], we also found a very strong correlation (R^2^ = 0.99) between the levels of HER2 expression and E_max_.

### 3.10. Influence of Assay Buffer, Target Cell Plating Method, and Treatment Time on the ADCC Activity of Trastuzumab

**Figure 7:**
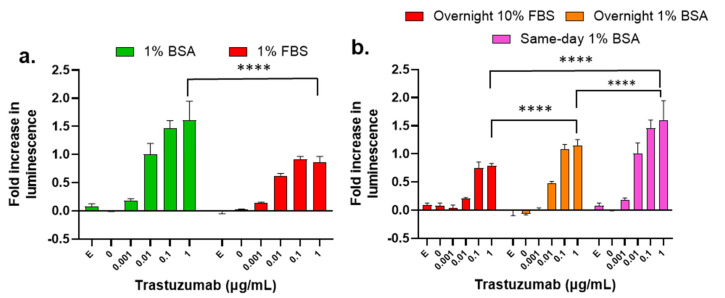
Influence of assay buffer, target cell plating method, and treatment time on the measurement of ADCC of trastuzumab. (**a**) Luminescence fold changes from the LDH ADCC assay using RPMI-1640 with 1% BSA or 1% FBS. Data were analyzed with GraphPad Prism using two-way ANOVA with Šídák’s multiple comparisons test. *p* value < 0.0001 for 1% BSA vs. 1% FBS; (**b**) Luminescence fold changes using the LDH ADCC assay performed with either overnight or same-day culture of SKBR-3 target cells plated with 1% BSA or overnight culture of SKBR-3 in 10% FBS. Data were analyzed with GraphPad Prism using two-way ANOVA with Tukey’s multiple comparisons test. **** *p* < 0.0001. SKBR-3 cells were treated with trastuzumab (0–1 µg/mL) and NK cells with E/T ratio 5:1, and the killing of SKBR-3 was measured with the LDH ADCC assay, where “E” stands for effector-cell-only control.

During our examination, we found that other ADCC assay parameters can also affect assay performance, such as assay buffer and target cell plating method. For example, 1% bovine serum albumin (BSA) as an additive to the assay buffer caused a higher S/N ratio (fold increase in luminescence) than the assays performed using 1% fetal bovine serum (FBS) as an additive (Figure 7a). Therefore, we used 1% BSA as the standard additive for all our ADCC assays. Next, we tested the influence of the target cell plating method on the S/N ratio of the ADCC assay. Three different plating approaches were assessed and compared to identify changes in the dose-dependent ADCC activity of trastuzumab. The differences in plating included SKBR-3 cells seeding one day prior to the assay with 10% FBS and 1% BSA, and on the same day as the assay with 1% BSA. All treatments were performed in 1% BSA regardless of plating method. These data indicate that same-day 1% BSA generally provides the best S/N ratio, and overnight 10% FBS provides lower S/N ratio (Figure 7b).

## 4. Discussion

ADCC is a complex process involving mAb binding to antigen on target cells and the Fc receptor on effector cells, and signal transduction to activate effector cells, resulting in polarization and release of cytotoxic or lytic molecules including pore-forming glycoproteins perforin and serine proteases granzymes into the target cells [41,44,45]. This in turn leads to DNA damage and cell apoptosis of the target cells.^33,34^ While the mechanisms of action of ADCC mediated by mAbs are well established, the assays employed for monitoring ADCC activity for product release and stability testing require continued development and/or refinement to properly assess and compare the product potency of newly developed therapeutic mAbs and biosimilars.

In this study, we optimize conditions for the LDH ADCC assay for trastuzumab and HER2 overexpressing target cells using NK cells or cryopreserved PBMCs as the effector cells. Optimization focused on defining critical parameters of assay to improve the quality of data, represented by the signal-to-noise ratio. Among the critical parameters tested in this study, we recommend culturing PBMCs overnight before NK isolation. The optimized ADCC functional assay medium is RPMI-1640 medium containing 1% BSA. Optimized treatment time is 24 h. SKBR-3 showed the best signal-to-noise ratio, and thus served as a good target cell line for this assay. E/T ratio between 5:1 and 20:1 provided good response, while there were still donor-to-donor variations depending on the effector cells used.

We report that the recovery cultivation of cryopreserved effector cells for 4–24 h was essential to restore the cytotoxic activity of NK or PBMCs cells in ADCC assays. Data support that the ideal condition to improve signal is to apply a 4 to 24 h recovery cultivation period for the freshly thawed cryopreserved PBMCs at 37 °C prior to isolating the NK cells for the ADCC assay. This optimized procedure to prepare NK cells from cryopreserved PBMCs will ensure the restoration of cytotoxic activity of NK cells with the best quality of ADCC potency data based on the results of our optimized assay system.

PBMCs contain a population of various cell types that include lymphocytes (NK cells, T cell, and B cells), monocytes, and dendritic cells. Although studies on cytokine production from PBMCs are usually carried out under stimulation from either IL-2 or phorbol 12-myristate 13-acetate (PMA)/Ionomycin, it was reported that spontaneous cytokine production, such as IL-6, could be observed [46], and that NK were directly activated by IL-6 [47]. Therefore, it is possible that spontaneous cytokine production by the subpopulation of different cell types in the PBMC pool during the recovery cultivation time increased, which may account for the potentiation of NK cells towards ADCC.

It has been previously reported that overnight cultivation of PBMCs could help restore the cytotoxic activity of the cryopreserved NK cell subpopulation [26,27]. However, the methodology of the recovery cultivation was not clearly described, and the mechanisms underlying the restoration of cytotoxic activity of NK cells were not investigated. In this study, alterations to cellular mechanisms during the recovery cultivation that can potentiate NK cells in ADCC assay were studied. Our data support that CD69, a classical early marker of NK cell activation [48,49,50]^,^ was drastically upregulated during the recovery cultivation period. This upregulation of CD69 was consistent with the restoration of trastuzumab-mediated ADCC activity and increase in protein expression and/or secretion of perforin, granzyme B, TNFα, and IFNγ. CD69 is a stimulatory receptor for NK cells and required for activated NK-cell-mediated killing [51]. Therefore, we speculate that the CD69 upregulation that occurs during the recovery cultivation period may lead to the NK cells activation process, which subsequently would enhance ADCC activity mediated by mAbs.

Human NK cells include two major subsets, the CD56^dim^ subset and CD56^bright^ subset [52]. The CD56^dim^ subset is believed to be potently cytotoxic and mediate ADCC activity, whereas the CD56^bright^ subset is generally thought to be more proliferative and less cytotoxic [52]. Our data support that a recovery cultivation period for freshly thawed PBMCs demonstrated a simultaneous downregulation of both CD56 and CD16 (Figure 4e). CD16 binds to the Fc portion of trastuzumab to mediate signal transduction, which leads to cytokine production and cytotoxic activity. Previous reports have shown a strong correlation between activation of CD56^dim^ NK cells by cross-linking CD16 with antibodies and a loss of CD16 expression with increased IFNγ production [53]. It was also reported that shedding of CD16 occurred during NK activation, helping detachment of NK cells from target cells, thus improving immune response by boosting serial engagement of target cells [54]. Our data support that the recovery cultivation of cryopreserved PBMCs appears to functionally mimic activation of NK cells induced by cross-linking CD16 with antibodies. On the other hand, CD56 is reportedly an important marker and recognition receptor on NK cells, and a similar downregulation of CD56 on NK cells was also reported upon pathogen contact [55]. We assume that this simultaneous reduction in CD16 and CD56 on NK cell surface, or the emergence of a new subset [CD56^dim^/CD16^dim^] subpopulation in NK cells (Figure 4e) during recovery cultivation, may be a consequence of the activation of NK cells after cryopreservation. Therefore, we speculate that recovery cultivation of NK cells within the PBMC population after the freeze–thaw cycle is an essential process that will enable NK cells to exert maximum activity, induce the secretion of perforin, granzyme B, IFNγ, and TNFα during the ADCC process, and consequently improve the consistency and quality of data obtain from the ADCC assay. We also found a delayed cell killing signal from unrecovered NK cells (comparing 24 h and 48 h data in Figure 1c, gray bar). This suggests that NK cells isolated from immediately thawed PBMCs could still be activated during the process of ADCC assay, presumably via interaction between antibody-loaded target cells and NK cells. This is different from the mechanism by which NK cells are activated during the recovery culturing of the PBMCs.

Additional novel observations were also identified in this study. The selection of the target cell line was identified as an important parameter for the ADCC assay. We screened three cancer cell lines (SKBR-3, BT-474, and NCI-N87) with the highest HER2 expression among cells tested. SKBR-3 cell line provides the highest S/N ratio or E_max_, significantly higher than BT-474 and NCI-N87 in the LDH ADCC assay. The ADCC reporter assay detects antibody-dependent activation of the reporter cells, and the differences between SKBR-3 and the other two cell lines were less significant. The rankings of EC_50_ values were not the same among different target cell lines in these two assays, with SKBR-3 being the most sensitive (lowest EC_50_) in the LDH ADCC functional assay, and NCI-N87 being the most sensitive (lowest EC_50_) in the reporter gene assay. The sensitivity order of these three cell lines represented by EC_50_ values in the reporter gene assay appeared consistent with the order of HER2 expression levels (NCI-N87 > BT-474 > SKBR-3) as determined by immunoblotting. However, the order of sensitivity in the LDH ADCC assay was inconsistent with the order of HER2 expression level. The reason underlying the sensitivity to LDH ADCC assay among HER2-overexpressed cell lines remains to be further investigated. Our data also support that 1% BSA as ADCC buffer additive increases the S/N ratio as compared to buffer that uses 1% FBS as an additive. Considering that FBS is a complex mixture that contains BSA and other components such as hormones, transport proteins, and growth factors with noncontrolled variability between lots, it is hard to predict the effect of each component. The use of 1% BSA as a raw material may improve control of a critical component of the ADCC assay and may improve data consistency.

The results obtained from this study are not only helpful for trastuzumab biosimilar development, but also generalizable to other therapeutic mAbs with ADCC as the major MOA, using cryopreserved PBMCs or NK cells to develop ADCC potency assays.

## Data Availability

The original contributions presented in the study are included in the article/supplementary material, further inquiries can be directed to the corresponding author/s.

## Supplementary Materials

The following supporting information can be downloaded at: https://www.mdpi.com/article/10.3390/cancers16132367/s1. Figure S1. Comparison of signal-to-noise ratio of LDH ADCC assay performed with different treatment time. Signal-to-noise ratio = relative luminescence [Emax]/Relative luminescence [0 µg/mL]. Data were analyzed with GraphPad Prism using one-way ANOVA analysis with Tukey’s post hoc test. *p* < 0.0001; Figure S2. Flow scheme showing two different recovery culturing methods used in Figure 2b,d and Figure 5c,d. The isolated PBMCs from leukopak are cryopreserved. After thawing, the PBMCs undergo either recovery cultivation for overnight at 37 °C or NK cells isolation immediately after thawing. NK cells isolated from the recovered PBMCs resulted in “R-PB”, while NK cells isolated from PBMCs immediately after thawing were designated as R-iso; Figure S3. Flow cytometry analysis of surface CD14, CD25, and CD107a expression on NK cells isolated from unrecovered (NR) PBMCs or from PBMCs after recovery cultivation for different time points (4 h, 24 h); Figure S4. Flow cytometry isotype controls for CD56, CD69 and CD16 antibodies; Figure S5. (a) Representative immunoblot showing HER2 expression in SKBR-3 with control and HER2 siRNA; (b) Dose-dependent curves of trastuzumab-mediated LDH ADCC assay using SKBR-3 cells transiently transfected with control (nontargeting) or HER2 siRNA. Target cells were treated with trastuzumab (0–1 µg/mL) and NK cells with E/T ratio 20:1 for 24 h, and killing of SKBR-3 was measured with LDH ADCC assay; (c) Dose-dependent curves of trastuzumab-mediated ADCC activity using ADCC reporter assay in SKBR-3 cells. Target cells were treated with trastuzumab (0–5 µg/mL) and Promega ADCC reporter cells with E/T ratio 6:1, and luminescence was measured after 6 h.
