# Supplementary material for: Define Critical Parameters of Trastuzumab-Mediated ADCC Assays via Assay Optimization Processes, Focusing on the Impact of Cryopreserved Effector Cells on Assay Performance
Source: Cancers (Basel). 2024 Jun 27;16(13):2367. doi: 10.3390/cancers16132367 (PMC11240353; doi:10.3390/cancers16132367)
Supplement: Supplementary file 1 [file cancers-16-02367-s001.zip › cancers-2994157-supplementary.pdf]

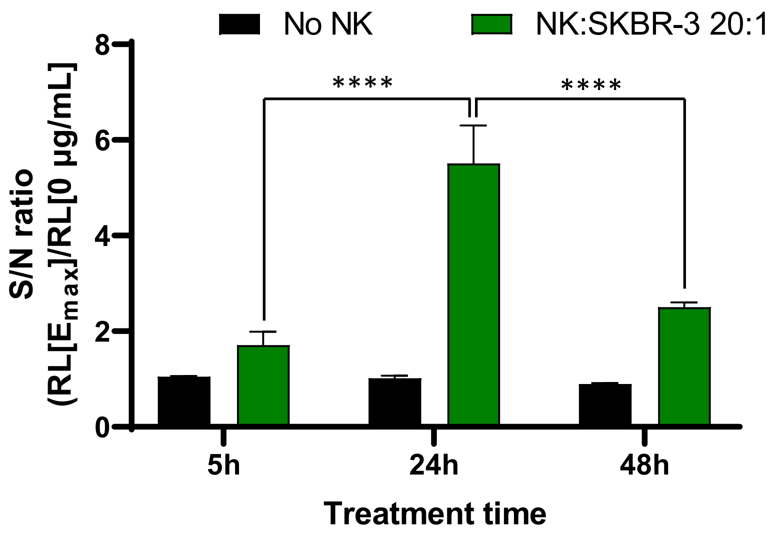

Figure S1. Comparison of signal-to-noise ratio of LDH ADCC assay performed with different treatment time. Signal-to-noise ratio = relative luminescence [Emax]/Relative luminescence [0 µg/mL]. Data were analyzed with GraphPad Prism using one-way ANOVA analysis with Tukey's post hoc test. $p < 0.0001$.

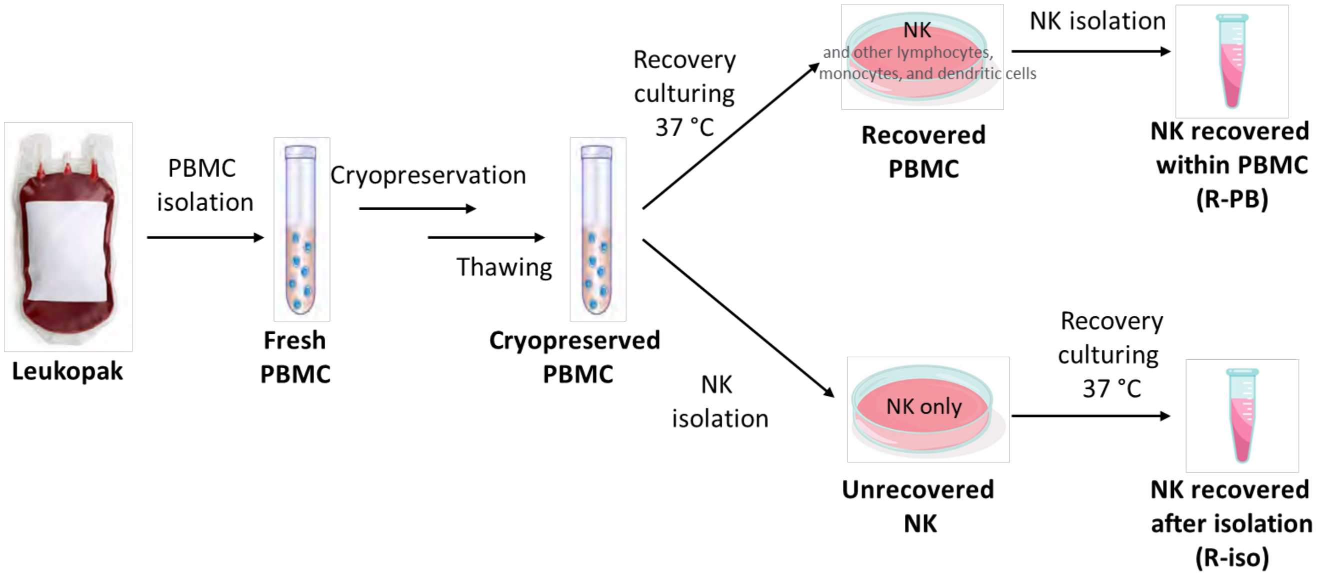

Figure S2. Flow scheme showing two different recovery culturing methods used in Figure 2b,d and Figure 5c,d. The isolated PBMCs from leukopak are cryopreserved. After thawing, the PBMCs undergo either recovery cultivation for overnight at 37 °C or NK cells isolation immediately after thawing. NK cells isolated from the recovered PBMCs resulted in “R-PB”, while NK cells isolated from PBMCs immediately after thawing were designated as R-iso.

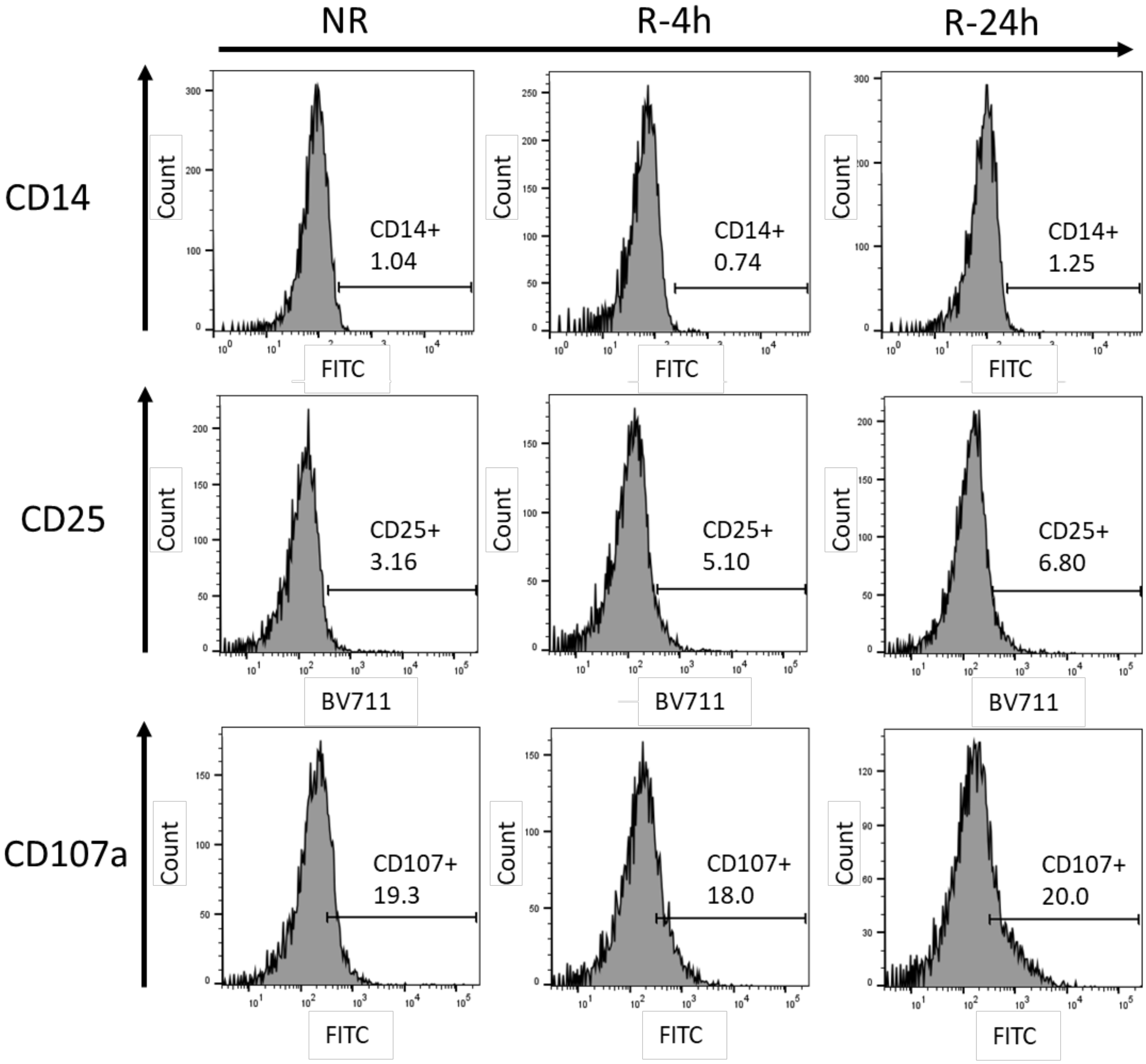

Figure S3. Flow cytometry analysis of surface CD14, CD25, and CD107a expression on NK cells isolated from unrecovered (NR) PBMCs or from PBMCs after recovery cultivation for different time points (4 h, 24 h).

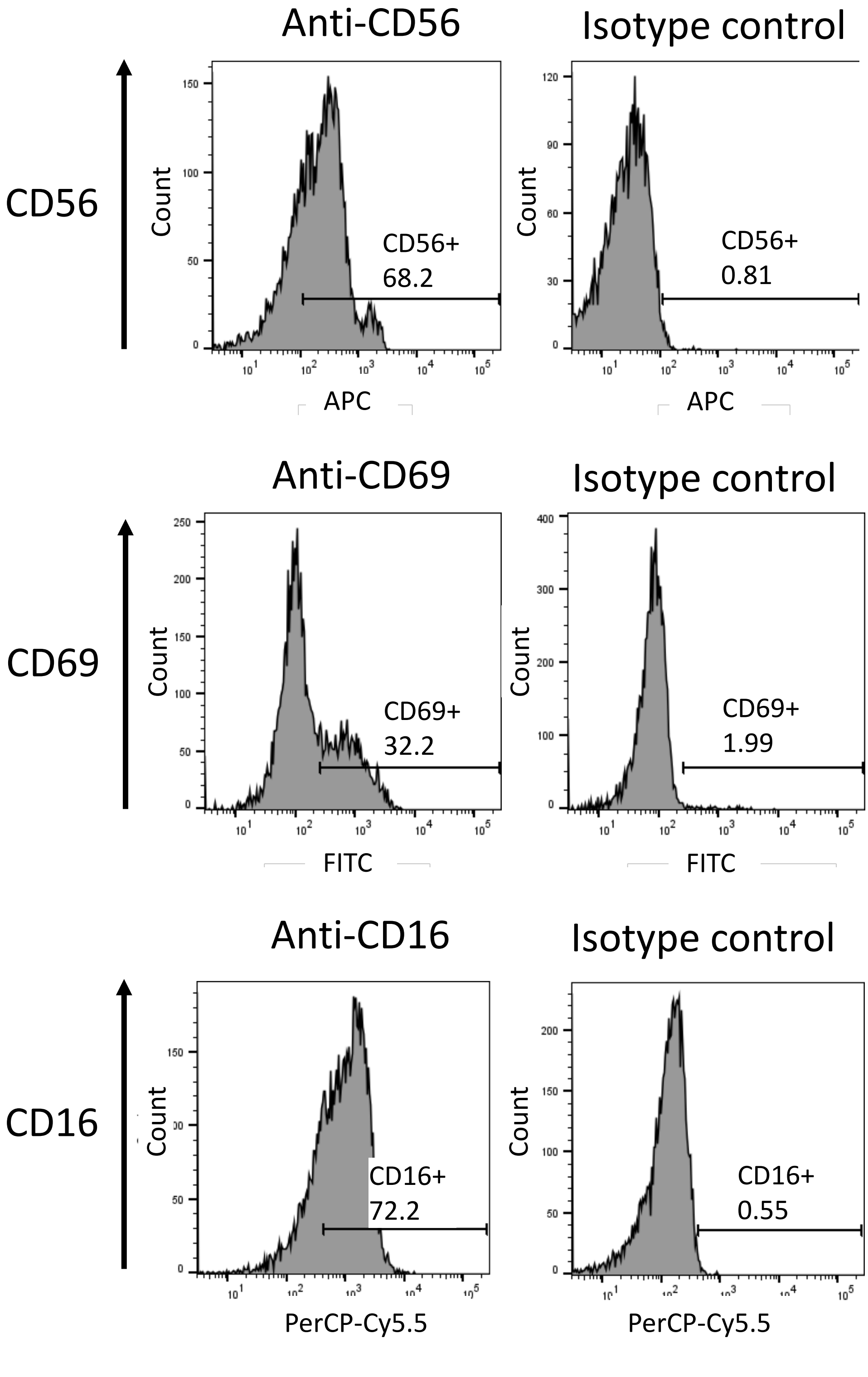

Figure S4. Flow cytometry isotype controls for CD56, CD69 and CD16 antibodies.

a.

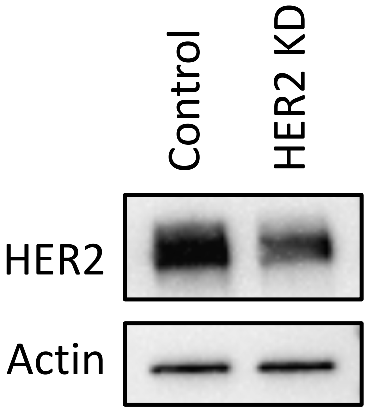

b.

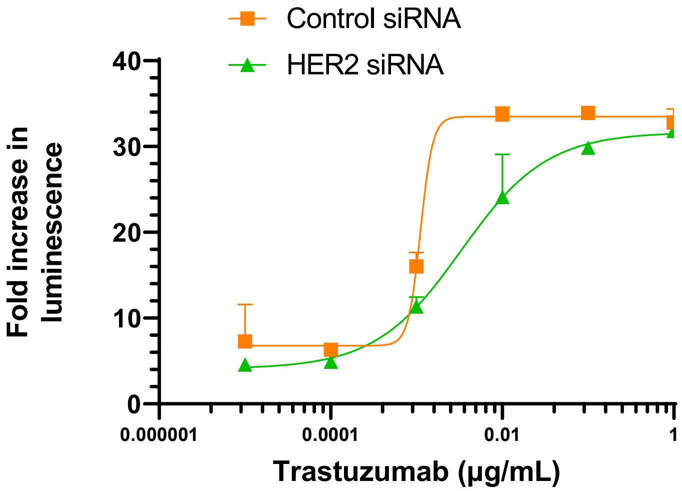

LDH ADCC Assay

	E_{max} (Fold increase)	EC_{50} (ng/mL)
Control	33.5	1.1
HER2 KD	31.7	3.3

c.

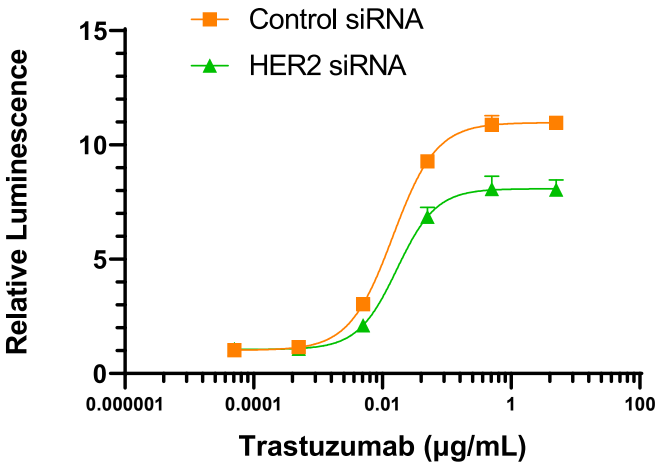

ADCC Reporter Assay

	E_{max} (Relative Luminescence)	EC_{50} (ng/mL)
Control	11.0	14.5
HER2 KD	8.1	16.8

Figure S5. (a) Representative immunoblot showing HER2 expression in SKBR-3 with control and HER2 siRNA; (b) Dose-dependent curves of trastuzumab-mediated LDH ADCC assay using SKBR-3 cells transiently transfected with control (nontargeting) or HER2 siRNA. Target cells were treated with trastuzumab (0–1 μg/mL) and NK cells with E/T ratio 20:1 for 24 h, and killing of SKBR-3 was measured with LDH ADCC assay; (c) Dose-dependent curves of trastuzumab-mediated ADCC activity using ADCC reporter assay in SKBR-3 cells. Target cells were treated with trastuzumab (0–5 μg/mL) and Promega ADCC reporter cells with E/T ratio 6:1, and luminescence was measured after 6 h.
